# Gap-less and haplotype-resolved genomes of two *Hippophae rhamnoides* subspecies: *Hippophae rhamnoides* subsp. *mongolica* and *Hippophae rhamnoides* subsp. *sinensis*

**DOI:** 10.1093/hr/uhaf299

**Published:** 2025-11-05

**Authors:** Zhi-Wei Wang, Ye Zhao, Peng Li, Longxin Wang, Kai-Hua Jia, Cheng-Jiang Ruan

**Affiliations:** Institute of Plant Resources, Key Laboratory of Biotechnology and Bioresources Utilization, Ministry of Education, Dalian Minzu University, Dalian, China; State Key Laboratory of Tree Genetics and Breeding, National Engineering Research Center of Tree Breeding and Ecological Restoration, College of Biological Sciences and Technology, Beijing Forestry University, Beijing, China; State Forestry and Grassland Administration Key Laboratory of Silviculture in Downstream Areas of the Yellow River, College of Forestry, Shandong Agricultural University, Taian, Shandong, China; School of Biological Science and Technology, University of Jinan, Jinan, China; Institute of Crop Germplasm Resources, Shandong Academy of Agricultural Sciences, Jinan, China; Institute of Plant Resources, Key Laboratory of Biotechnology and Bioresources Utilization, Ministry of Education, Dalian Minzu University, Dalian, China

Dear Editor,

Seabuckthorn (*Hippophae rhamnoides*; 2*n* = 2*x* = 24), a member of the Elaeagnaceae family, is native to Asia and Northwestern Europe. This species is renowned for its exceptional resilience to extreme environmental conditions, including temperatures ranging from −40°C to +40°C, drought, and waterlogging, making it highly adaptable for cultivation. Its berries are rich in nutrients and bioactive compounds, notably high levels of flavonoids and vitamin C (Vc), which contribute to its widespread domestication and global cultivation. Recent advancements in sequencing technologies and genome assembly methods have enabled the successful decoding of numerous chromosome-level genomes, some even achieving gap-less or gap-free resolution [1]. Notably, several seabuckthorn genomes have been assembled at the chromosome level, including *Hippophae rhamnoides* subsp. *mongolia* (*H. r.* subsp. *mongolica*) [2], *Hippophae tibetana* [3], *H. rhamnoides* subsp*. sinensis (H. r.* subsp. *sinensis)* [4], and *Hippophae gyantsensis*[5]. However, the presence of numerous gaps and unplaced contig sequences poses significant challenges, potentially impacting downstream data analysis and functional research.

In this study, we assembled gap-less, haplotype-resolved genomes of two representative seabuckthorn subspecies *H. r.* subsp. *mongolica* which is characterized by large fruits, high yield, high oil content, and sparse thorns; and *H. r.* subsp. *sinensis*, which exhibits strong stress resistance, rapid growth, and high Vc content. For *H. r.* subsp. *mongolica*, we sequenced 37 Gb of PacBio HiFi reads (30× coverage), 67 Gb of Oxford Nanopore Technologies (ONT) reads (54× coverage), and 73 Gb of Hi-C reads. For *H. r.* subsp. *sinensis*, a similar strategy was employed, yielding 37 Gb of PacBio HiFi reads (35× coverage), 61 Gb of ONT reads (60× coverage), and 83 Gb of Hi-C reads. We utilized hifiasm (v0.19.8-r602) to assemble PacBio HiFi and ONT reads into contigs. Hi-C reads were subsequently aligned to the haplotype-resolved assemblies using Juicer (v1.6), and chromosome scaffolding using 3D-DNA (v180419). Manual inspection and correction of chromosome boundaries and misassemblies were performed using Juicebox (v1.11.08). Gaps were filled using quarTeT (v1.2.5) based on HiFi reads. For HiFi reads aligning to chromosome ends, we reassembled these reads into contigs to extend the chromosome lengths as much as possible, aiming to capture the complete telomere sequences. Additionally, GetOrganelle (v1.7.7.1) was employed to assemble the chloroplast and mitochondrial genomes.

**Figure 1:**
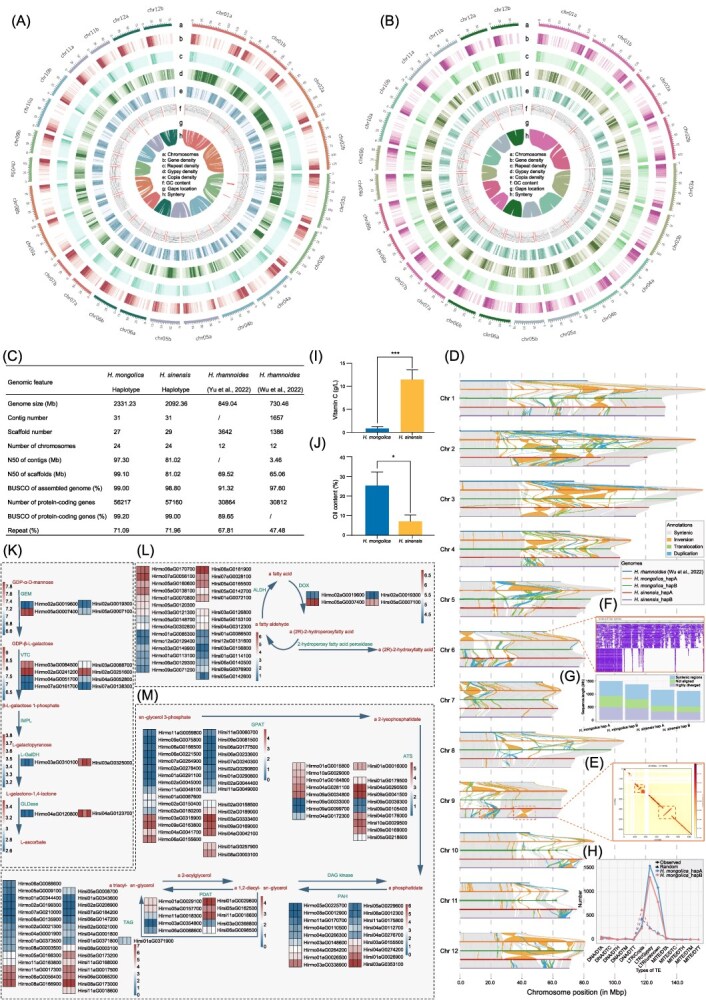
Genomic and transcriptomic profiling of *H. r.* subsp. *mongolica* and *H. r.* subsp. *sinensis*. (A, B) Circos plots depicting the genomic features of *H. r.* subsp. *mongolica* (A) and *H. r.* subsp.*sinensis* (B). From outermost to innermost: (a) chromosomes; (b) gene density; (c) repeat density; (d) Gypsy element density; (e) Copia element density; (f) GC content, with red lines at chromosome ends indicating that telomeres were assembled; (g) gaps location (h) synteny. For all tracks except chromosomes, darker shades indicate higher feature density. In the synteny track, lines represent collinear relationships between homologous regions of haplotypes A and B within each species, with alignments exceeding 100 kb. (C) Comparison of genome assembly metrics for the newly sequenced *H. r.* subsp. *mongolica* and *H. r.* subsp. *sinensis* genomes relative to a previously published reference. (D) Synteny and structural variation across haplotype-resolved assemblies of five *Hippophae* genomes. Synteny analysis was performed using SyRI, comparing each pair of haplotype-resolved assemblies to characterize structural variation. The horizontal axis shows the twelve reference chromosomes (chr1–chr12) with positions in Mb. Each track represents alignments between the query genomes and the reference, with structural variations categorized as: syntenic regions (grey), inversions (orange), translocations (green), and duplications (blue). (E) Hi-C interaction heatmap between the two phased haplotypes (chr9a and chr9b) of chromosome 9 in *H. r.* subsp. *sinensis.* The diagonal represents self-interactions within each haplotype, with high interaction frequencies (red) indicating close physical proximity in 3D space. (F) IGV visualization of HiFi read alignments on *H. r.* subsp. *mongolica* chr06a (8617048–9 607 932 bp). (Top) HiFi reads from *H. r.* subsp. *rhamnoides* [4] aligned to the *H. r.* subsp. *mongolica* haplotype A assembly. (Bottom) HiFi reads from *H. r.* subsp. *mongolica* aligned to its own haplotype A assembly. (G) Stacked bar chart summarizing variation types from SyRI statistics. (H) Number of inversion breakpoints (150 bp each) overlapping with TEs in the two haplotypes of *H. r.* subsp. *mongolica*. The solid line represents the observed pattern, and the dashed line represents the randomized pattern (1000 shuffles). HapA and HapB are shown in blue and red, respectively. (I) Vc content in the fruit pulp of *H. r.* subsp. *mongolica* and *H. r.* subsp. *sinensis*. (J) Seed oil content in *H. r.* subsp. *mongolica* and *H. r.* subsp. *sinensis*. Data are presented as mean ± SD (*n* = 3); statistical significance was assessed using a Student's *t*-test (*P* < 0.05). (K-M) Heat maps of transcript abundance (log₂(TPM)) for genes involved in (K) Vc biosynthesis (log₂(TPM) ≥ 1) and (L) triacylglycerol (TAG) biosynthesis and (M) fatty acid metabolism, with gene IDs beginning with ‘Hirmo’ and ‘Hirsi’ denoting *H. r.* subsp. *mongolica* and *H. r.* subsp. *sinensis*, respectively. Each row corresponds to either a pair of orthologous genes or a subspecies-specific gene detected only in one subspecies without a homolog in the other. Only genes with TPM ≥ 1 in at least one sample are shown.

After removing redundant sequences, 12 pairs of chromosomes along with chloroplast and mitochondrial genomes were successfully assembled. The genomes of *H. r.* subsp. *mongolica* and *H. r.* subsp. *sinensis* contained 4 and 2 gaps, respectively, with 38 and 42 telomeres assembled (Fig. 1A-B). The haplotype-resolved assemblies for *H. r.* subsp. *mongolica* and *H. r.* subsp. *sinensis* were 2.33 Gb and 2.09 Gb, respectively, with contig N50 values of 97 Mb and 81 Mb. For *H. r.* subsp. *mongolica*, 99.55% of the PacBio HiFi reads mapped back to the assembly, and 99.76% of the genome was covered by at least 5× HiFi depth. The final genome assemblies exhibited high completeness, with BUSCO (v5.8.2) analysis in genome mode yielding 99.0% complete BUSCOs for *H. r.* subsp. *mongolica* and 98.8% for *H. r.* subsp. *sinensis*. Hi-C contact maps for both assemblies exhibited clear diagonal blocks without obvious mis-joins, indicating high chromosomal accuracy. These results demonstrate that we have achieved two gap-less haplotype-resolved genomes assemblies. In addition, we assessed phasing quality using multiple complementary approaches, including *K*-mer spectrum analysis and quantitative evaluation of switch errors. *K*-mer analysis verified the accuracy of the assembly results, indicating that the sequence differences between haplotypes were clearly distinguished without significant contamination or mosaicism. Switch-error rates were 0.8% for *H. r.* subsp. *mongolica* and 0.6% for *H. r.* subsp. *sinensis*, both well below the 1% threshold commonly accepted for high-quality phased assemblies. Compared to previously reported seabuckthorn genomes, our assemblies exhibit markedly improved contiguity, with contig N50 values increased by over 20-fold and a substantial reduction in scaffold numbers (Fig. 1C).

Using EDTA (v1.9.9) followed by RepeatMasker (v4.1.8), we annotated 71.09% of the *H. r.* subsp. *mongolica* genome and 71.96% of the **H. r*.* subsp. *sinensis* genome as repetitive. In both assemblies, long terminal repeats (LTRs) dominate: they account for 44.74% of *H. r.* subsp. *mongolica* and 46.58% of *H. r.* subsp. *sinensis*. Within the LTR fraction, LTR/Gypsy elements are the largest component—23.44% in *H. r.* subsp. *mongolica* and 12.56% in *H. r.* subsp. *sinensis*. The marked difference in LTR/Gypsy representation is explained by the proportion of unclassified LTRs: 10.67% in *H. r.* subsp. *mongolica* versus 22.82% in *H. r.* subsp. *sinensis*.

Based on homology evidence, transcript evidence and *ab initio* predictions, we annotated 56 217 protein-coding genes in *H. r.* subsp. *mongolica* and 57 160 in *H. sinensis*. In addition to these protein-coding genes, the *H. r.* subsp. *mongolica* assembly contains 791 rRNA, 1460 tRNA and 4897 ncRNA genes, whereas the *H. r.* subsp*. sinensis* assembly contains 1885 rRNA, 1200 tRNA and 5319 ncRNA genes. BUSCO assessment of the annotated protein-coding gene sets revealed completeness values of 99.2% for *H. r.* subsp. *mongolica* and 99.0% for *H. r.* subsp. *sinensis*. Functional annotation indicated that 98.05% of *H. r.* subsp. *mongolica* proteins and 98.15% of *H. r.* subsp. *sinensis* proteins could be assigned putative functions.

Each haplotype in our assemblies is approximately 1.5 times larger than the previously published chromosome-level assemblies of related subspecies (Fig. 1C). To explore the origin of these additional sequences, we conducted whole-genome comparisons using SyRI (V1.6) between the *H. rhamnoides* reference genome [4] and all haplotypes of *H. r.* subsp. *mongolica* and *H. r.* subsp. *sinensis*. The analyses revealed extensive structural rearrangements across all 12 chromosomes, dominated by frequent inter-chromosomal translocations and intra-chromosomal inversions (Fig. 1D). To further validate the authenticity of the structural inversions identified by SyRI, we examined the Hi-C interaction patterns between the two haplotypes of *H. r.* subsp. *sinensis*, which revealed off-diagonal contact signals consistent with the predicted inversion events (Fig. 1E). Compared with previously published assemblies [2, 4], each haplotype in our genomes contains substantially more sequences. To verify that these additional sequences, which were missing in earlier assemblies [4, 5], are genuine rather than artifacts of sequencing or assembly errors, we mapped publicly available HiFi reads of *H. rhamnoides* [4] and found no support for the expanded regions, whereas our own HiFi data provided full coverage, confirming that these sequences represent authentic genomic differences (Fig. 1F). More than 40% of aligned regions exhibited non-syntenic relationships, with inversions alone affecting 147–210 Mb of sequence in each haplotype comparison. In addition, non-syntenic or unaligned regions accounted for 28.3% to 48.4% of each query genome, highlighting substantial haplotype-specific variation, interspecies divergence, and the expansion of repetitive or structurally complex genomic regions. For instance, *H. r.* subsp. *sinensis* hapB contained 208 Mb of sequences absent from *H. rhamnoides*, while *H. r.* subsp. *mongolica* hapA carried more than 426 Mb of such regions (Fig. 1G).

To investigate the potential forces underlying these large-scale structural variations, we next examined the contribution of transposable elements (TEs). Genome-wide enrichment analysis revealed that LTR/Gypsy elements are non-randomly distributed, showing significant accumulation within structurally rearranged regions compared with randomized expectations (Fig. 1H). Notably, LTR/Gypsy elements were disproportionately enriched at chromosomal breakpoints (hapA: 2360; hapB: 1810) relative to broader inversion regions (hapA: 1547; hapB: 1308). These results suggest that LTR/Gypsy elements have actively mediated chromosome breakage and rearrangements, thereby playing a key role in shaping the distinct genome architectures of *H. r.* subsp. *mongolica* and *H. r.* subsp. *sinensis*.

The two subspecies exhibited notable differences in fruit pulp Vc content and seed oil content (Fig. 1I and J). **H. r*.* subsp. *sinensis* accumulates significantly higher levels of Vc in the fruit pulp (Fig. 1I). To elucidate the molecular basis of this trait, we examined the ascorbate biosynthesis pathway. Several key genes, including GDP-l-galactose phosphorylase (VTC) (*Hirsi04aG0052800*), l-galactose dehydrogenase (l-GalDH) (*Hirsi03aG0325000*), and l-galactono 1,4-lactone dehydrogenase (GLDase) (*Hirsi04aG0123700*), exhibited markedly higher expression in *H. r.* subsp. *sinensis* relative to *H. r.* subsp. *mongolica*  **(**Fig. 1K), consistent with the elevated Vc content. These genes exhibited statistically significant differential expression between the two species (*t*-test, *P* < 0.05), suggesting that their upregulation may contribute to the observed species-specific difference in Vc accumulation (Fig. 1I).

Conversely, *H. r.* subsp. *mongolica* exhibited significantly higher seed oil content than *H. r.* subsp. *sinensis* (Fig. 1I). Transcriptomic profiling of triacylglycerol (TAG) and fatty acid biosynthesis pathways revealed enhanced expression of an acyl-CoA:diacylglycerol acyltransferase (*Hirmo06aG0056400*), a key enzyme in TAG synthesis, and a species-specific glycerol-3-phosphate O-acyltransferase (GPAT) (*Hirmo09aG0163800*) in *H. r.* subsp.*mongolica* (Fig. 1L). In addition, two fatty aldehyde dehydrogenase (ALDH) (*Hirmo07aG0056100* and *Hirmo05aG0138100*), along with a species-specific ALDH (*Hirmo05aG0120300*), were upregulated in the fatty acid pathway (Fig. 1M). Statistical tests confirmed that these genes are significantly more highly expressed in *H. r.* subsp. *mongolica* compared with *H. r.* subsp. *sinensis* (*t*-test, *P* < 0.05), supporting their contribution to the elevated seed oil content (Fig. 1J).

In summary, we present two gap-less, haplotype-resolved genomes of *H. r.* subsp. *mongolica* and *H. r.* subsp. *sinensis*, alongside tissue-specific transcriptomic data linked to key fruit quality traits. These resources not only provide a high-resolution view of genome architecture and gene content, but also offer mechanistic insights into the species-specific accumulation of Vc and seed oil. Together, they form a foundational platform for evolutionary studies and targeted breeding in seabuckthorn.

## Data Availability

The raw genomic sequencing data have been deposited in the China National Center for Bioinformation (CNCB) under accession number CRA024756. The whole-genome assemblies of *H. r.* subsp. *mongolica* and **H. r*.* subsp. *sinensis* are available under accession numbers GWHFSQC00000000.1 and GWHFSQD00000000.1, respectively. Transcriptome datasets from fruit pulp and seed tissues of both subspecies have been archived in the CNGB Sequence Archive (CNSA) under project accession CNP0007442.
